# Detection of ROS Induced Proteomic Signatures by Mass Spectrometry

**DOI:** 10.3389/fphys.2017.00470

**Published:** 2017-07-07

**Authors:** Brian McDonagh

**Affiliations:** Department of Physiology, School of Medicine, NUI GalwayGalway, Ireland

**Keywords:** redox modifications, sulfenic, nitrosylation, glutathionylation, tyrosine nitration, carbonylation, targeted proteomics

## Abstract

Reversible and irreversible post-translational modifications (PTMs) induced by endogenously generated reactive oxygen species (ROS) in regulatory enzymes and proteins plays an essential role in cellular signaling. Almost all cellular processes including metabolism, transcription, translation and degradation have been identified as containing redox regulated proteins. Specific redox modifications of key amino acids generated by ROS offers a dynamic and versatile means to rapidly alter the activity or functional structure of proteins in response to biochemical, environmental, genetic and pathological perturbations. How the proteome responds to these stimuli is of critical importance in oxidant physiology, as it can regulate the cell stress response by reversible and irreversible PTMs, affecting protein activity and protein-protein interactions. Due to the highly labile nature of many ROS species, applying redox proteomics can provide a signature footprint of the ROS species generated. Ideally redox proteomic approaches would allow; (1) the identification of the specific PTM, (2) identification of the amino acid residue that is modified and (3) the percentage of the protein containing the PTM. New developments in MS offer the opportunity of a more sensitive targeted proteomic approach and retrospective data analysis. Subsequent bioinformatics analysis can provide an insight into the biochemical and physiological pathways or cell signaling cascades that are affected by ROS generation. This mini-review will detail current redox proteomic approaches to identify and quantify ROS induced PTMs and the subsequent effects on cellular signaling.

## Introduction

The specific reduction or oxidation (redox) of critical enzymes/proteins as a result of endogenously generated reactive oxygen or reactive nitrogen species (ROS/RNS) can alter the metabolic flux within a cell. Long term modifications of metabolic flux can have chronic effects resulting in metabolic disorders associated with conditions, such as Type 2 diabetes (Watson, 2014), cancer (Yuan et al., 2014), aging (Muller et al., 2007), and neurodegenerative diseases (Butterfield et al., 2012). Specific redox modifications offer a dynamic and versatile means to rapidly alter the activity or functional structure of proteins in response to stimuli. In many examples, these stimuli refer to ROS/RNS that are endogenously generated. Endogenous generation of ROS/RNS is essential for correct adaptation and signaling in normal cellular functioning including cell proliferation, metabolism, immune response, antioxidant defenses and in the adaptation and response to exercise (Schieber and Chandel, 2014). These small molecules can react with a range of enzymes in almost every cellular process including glycolysis, oxidative phosphorylation, amino acid biosynthesis, pentose phosphate pathway, autophagy, transcription, translation etc., (reviewed in Margaritelis et al., 2016).

Although generally referred to as ROS/RNS, this term covers a range of small molecules including superoxide radical, hydroxyl radical and hydrogen peroxide. Some of these (e.g., superoxide and hydroxyl radicals) are unstable, whereas others e.g., H_2_O_2_, are more freely-diffusible and can be relatively long-lived. The traditional view of ROS/RNS generation by electron leakage from the electron transport chain within the mitochondria has been expanded as their role as signaling molecules within the cell has developed. Two families of known endogenous producers of ROS/RNS are the NAD(P)H oxidase (NOX) family and nitric oxide synthases (NOS), which predominantly generate superoxide (${\text{O}}_{2}^{-\cdot }$) and nitrogen monoxide (NO), respectively. Indeed, NO and ${\text{O}}_{2}^{-\cdot }$ produced by NOX and NOS may react and form another redox oxidant, peroxynitrite (ONOO^−^), these species of ROS/RNS have different chemical reactivity and kinetics, resulting in distinct post-translational modifications (PTMs) on target proteins with further downstream redox effects. ROS/RNS differ in their rates of diffusion and reactivity and in general are thought to be too reactive to produce long range signaling effects both intracellularly and between cells, therefore they are considered to have an effect localized to their site of generation, modifying susceptible and in some cases critical residues on proteins in their immediate vicinity (Winterbourn, 2015). Under normal physiological conditions ROS/RNS are no longer viewed as non-specific oxidation instruments but involved in a co-ordinated local response that is tightly regulated at all levels from generation to detoxification (Corcoran and Cotter, 2013).

A large number of protein PTMs are the result of either direct or indirect interactions of proteins with ROS/RNS that can result in both reversible and irreversible protein modifications. Identification of the modification on the protein targets may provide a signature footprint of the specific ROS/RNS species that was present. This review will outline the application of redox proteomic techniques for the identification and where possible the quantification in particular of reversible redox modifications on specific amino acid residues in proteins. A number of these techniques have been employed in known metabolic diseases using non-invasive clinical samples, such as blood and urine, to identify redox specific biomarkers of metabolic diseases. However, many of the more recent redox proteomic techniques originally developed in unicellular organisms and cell culture systems offer the opportunity to be translated into clinically relevant models of disease.

## Types of redox modifications

Redox modifications on key metabolic processes can alter a wide variety of downstream protein targets, influencing key regulators of distinct PTMs, such as phosphorylation, acetylation and ubiquitination. These include components that control metabolic rate including AMP-activated protein kinase, protein kinase C, adenylate kinase, mammalian target of rapamycin and pyruvate kinase (Corcoran and Cotter, 2013). In particular redox modifications of Cysteine (thiol) residues have been extensively studied and can result in reversible and irreversible modifications with effects on protein function (Table 1). The location of a redox sensitive Cysteine within a protein can play an important part with regard to its redox state. Cysteine residues are one of the least abundant amino acids and have the most extreme conservation pattern within proteins, highly conserved when they form part of an active site or involved in co-factor binding and poorly conserved otherwise (Marino and Gladyshev, 2010).

**Table 1:** Common ROS/RNS induced modifications.

**Common ROS/RNS modification**	**ΔMass**	**Selective reduction**	**Probes/Antibody**	**References**
Disulfide bond formation (S-S-)	2	Thioredoxin system	Directly by MS	Zhao et al., 2013
Glutathionylation (S-S-G)	305.3	Glutaredoxin system	BioGEE, Anti-PSSG	Ying et al., 2007; Sakai et al., 2012
Nitrosylation (SNO)	28.99	Cu/Ascorbate	Anti-SNO	Jaffrey et al., 2001; Salanova et al., 2013
Sulfenylation (SOH)	15.99	Sodium Arsenite	Dimedone based	Saurin et al., 2004; Nelson et al., 2010
Sulfinic acid (SO_2_H)	31.99	Sulfiredoxin^*^	NO-Bio	Wagner et al., 2002; Lo Conte et al., 2015; Paulech et al., 2015
Sulfonic acid (SO_3_H)	47.99	–	Directly by MS	Wagner et al., 2002; Paulech et al., 2015
3-Nitrotyrosine	44.98	Sodium dithionite	Anti-3NT	Ghesquiere et al., 2006
Carbonylation (C = O)	^**^		Hydrazide chemistry	Fedorova et al., 2014; Havelund et al., 2017

Table includes the mass shift that is accompanied on the amino acid where available by the redox modification, specific reductants of the modification, available probes and antibodies selective for the particular redox modification.

* Sulfinic acids are generally reported to be irreversible apart from the selective reduction of 2-Cys peroxiredoxins by sulfiredoxin (Biteau et al., 2003).

** *Variable and dependent on amino acid modified*.

## Cysteine or thiol group modifications

The formation of sulfenic acids on protein thiols (-SOH) generally occurs by the reaction of Cysteine thiol(ate)s with H_2_O_2_ (although alkyl hydroperoxides and peroxynitrite may also play a role) and the reactivity of the Cysteine is strongly dependent on the ionization state of the thiol (Poole et al., 2004). Sulfenic acids are highly reactive and unstable and are considered intermediaries in the formation of more stable disulfide bonds when they react with a second thiol (Claiborne et al., 1999). Nevertheless, proteins containing stable sulfenates have been reported (Saurin et al., 2004; Charles et al., 2007; Salsbury et al., 2008) and it is thought that this stability depends on aspects of the protein microenvironment: local hydrogen bonds; lack of solvent accessibility to modified Cysteines; absence of nearby reduced Cysteines and amines (Claiborne et al., 1999; Salsbury et al., 2008). The importance of sulfenic acids in the formation and hydrolysis of disulfide bonds has been discussed in depth (Claiborne et al., 1999; Poole et al., 2004; Gallogly and Mieyal, 2007) and it is generally considered that the local environment allows sulfenic acids to react with proximal thiols, amines or GSH. Some of the techniques used to date to detect sulfenic acids include biotin labeled dimedone and fluorescent dimedone or using sodium arsenite for selective reduction (Saurin et al., 2004; Charles et al., 2007; Poole et al., 2007). Further oxidation of sulfenic acids to the generally irreversible sulfinates (-SO_2_H) or sulfonates (-SO_3_H) can occur, although sulfinic acid formation in 2-Cys Peroxiredoxins can be specifically reduced by Sulfiredoxin (Biteau et al., 2003). Sulfinic/sulfonic acid formation are stable modifications so could potentially be directly identified by including the change in mass as a variable modification. Due to their low relative abundance it can be difficult to characterize under normal conditions, although sensitivity can be increased using strong cation exchange (Paulech et al., 2015).

A further fate of sulfenic acids is sulfenylamide (Cys-S-N-R) formation, which has been reported in protein tyrosine phosphatase when a sulfenic reacts with the main chain amide nitrogen of an adjacent Serine residue (Salmeen et al., 2003; van Montfort et al., 2003). This modification was reported to be reducible by GSH and thus may prevent the irreversible oxidation of the Cysteine residue (Salmeen et al., 2003). It is not yet clear if this modification represents a genuine redox response or if it is a side reaction of sulfenic acid reactivity.

## Disulfide bond formation

A secondary result of increased ROS is an increase in overall protein disulfide bond formation. The formation of inter- and intra-disulfides between Cysteines can act as a mechanistic control for the activity of sensitive proteins, and lead to activation or inactivation depending on the protein involved. High throughput analysis of proteins containing disulfide bonds in complex mixtures has utilized a top down MS approach for comparing non-reduced and reduced proteins where a mass shift of 2 Da is indicative of a disulfide bond (Zhao et al., 2013). The effect of disulfide formation is highly dependent on the position of the Cysteine involved, if it forms part of the active site, the disulfide may be part of the catalytic cycle or act as an “on-off” switch for the activity of the protein (Jones, 2008). Alternatively, the formation of disulfide bonds may allosterically regulate protein activity, where formation of disulfides may change the structure of the protein (Jones, 2008). Formation of disulfides is also part of cell signaling by activation or export, such as Yap-1 (Delaunay et al., 2000) and indirectly Nrf2 (Dinkova-Kostova et al., 2002). A recent report identified the formation of a “redox relay” between Prdx2 and STAT3 required for the shuttling of the transcription factor from the cytoplasm to the nucleus in response to elevated H_2_O_2_ concentrations (Sobotta et al., 2015). Cytoskeletal remodeling mediated through disulfide bonds, plays a key role in the “respiratory burst” during phagocytosis required for the elimination of pathogens which is also dependent on ROS production (Sakai et al., 2012). One of the most important ROS induced disulfide bonds is the reversible formation of glutathionylated or glutathiolated proteins, which have key signaling roles and there are a number of protein families, such as the glutathione S-transferases and glutaredoxin 1 and 2, involved in (de)glutathionylation of proteins. Glutathionylation can be directly detected by MS by an increase in the mass of proteins by 305 Da (Hashemy et al., 2007).

## S-nitrosylation

The effects of Nitric oxide (NO) on signaling and metabolic pathways is also attributed to S- nitrosylation (or nitrosation) on key Cysteine residues of specific proteins. The formation of S-nitrosylation on thiol groups can occur directly through interaction with NO, indirectly through ONOO^−^ and there are also reports of transfer of nitrosyl or transnitrosylation by the actions of proteins, such as thioredoxins (Benhar, 2015). S-nitrosylation on target proteins is considered an important mechanism for NO signaling transduction and there are a large number of articles identifying specific Cysteine residues as S-nitrosylation targets in a variety of cellular systems from plants to cardiovascular systems (Hess et al., 2005), however as this PTM is reversible and highly labile PTM it has been suggested as an intermediate in the formation of disulphide bonds (Wolhuter and Eaton, 2017). Identification of S-nitrosylation is generally performed using a combination of selective reduction of S-nitrosylation proteins and labeling with a more stable reagent (Jaffrey and Snyder, 2001).

## Tyrosine nitration

Nitration of tyrosine amino acids is considered a signature of excessive ONOO^−^ and/or NO generation, however the subset of Tyrosine residues available for nitration is generally considered to be low and consequently the number of proteins detected to be nitrated (Tyther et al., 2011; Batthyany et al., 2017). Interest in Tyrosine nitration in particular is due to the role of Tyrosine in phosphorylation/dephosphorylation signaling. There are a number of potential pathways leading to Tyrosine nitration although many of these may not be kinetically feasible within a biological system, it is generally thought that ONOO^−^ is responsible for Tyrosine nitration on a limited subset of proteins. The hydrophobicity of Tyrosine nitration has also limited its detection by MS/MS but it has been detected in a number of biological conditions, the nitration of Tyr34 in MnSOD has been well-described where ONOO- formation as a result of excessive ${\text{O}}_{2}^{-\cdot }$ and NO results in MnSOD inactivation and further cellular damage, reported in a number of disease states (MacMillan-Crow and Thompson, 1999; Redondo-Horcajo et al., 2010).

## Carbonylation

Irreversible modifications, are generally associated with permanent loss of protein function and may result in accumulation of damaged proteins, such as in atherosclerosis and Alzheimer's disease (Davies et al., 1999). The balance between the rate of oxidized protein accumulation and degradation is dependent on a number of factors including ROS levels and protease activities catalyzing their degradation. Protein carbonylation occurs when amino acid side-chains are modified into aldehyde and ketone groups which can lead to protein aggregation, inactivation or degradation (Levine et al., 1990). The number of carbonyl groups has been shown to correlate with levels of oxidative stress and hence protein damage (Shacter et al., 1994). Carbonylation of proteins is a good indicator of oxidative damage and has been intensively investigated in systems, such as mammalian tissues, cell and yeast cultures (Costa et al., 2002; Rabek et al., 2003). Accumulation of carbonyls has also been observed in several human pathologies including Alzheimer's and Parkinson's diseases (Floor and Wetzel, 1998; Conrad et al., 2000). The amino acids especially susceptible to oxidation and thus carbonyl modifications are Pro, Arg, Lys, and Thr. Carbonyl derivatives may also be produced by the oxidative cleavage of proteins by either the α-amidation pathway or by oxidation of glutamyl side chains leading to the formation of a peptide in which the N-terminal amino acid is blocked by an α-ketoacyl derivative (Berlett and Stadtman, 1997). The various reactive products produced during lipid peroxidation, such as 4-hydroxy-2-nonenal can introduce carbonyl groups by reacting with the nucleophilic side chains of Cys, His and Lys residues (Berlett and Stadtman, 1997). A number of recent methods for the purification and subsequent identification by MS of carbonylated proteins have been described that take advantage of the reaction of the carbonyl group with hydrazide (Havelund et al., 2017).

## Detection of ROS induced PTMs

**Figure 1:**
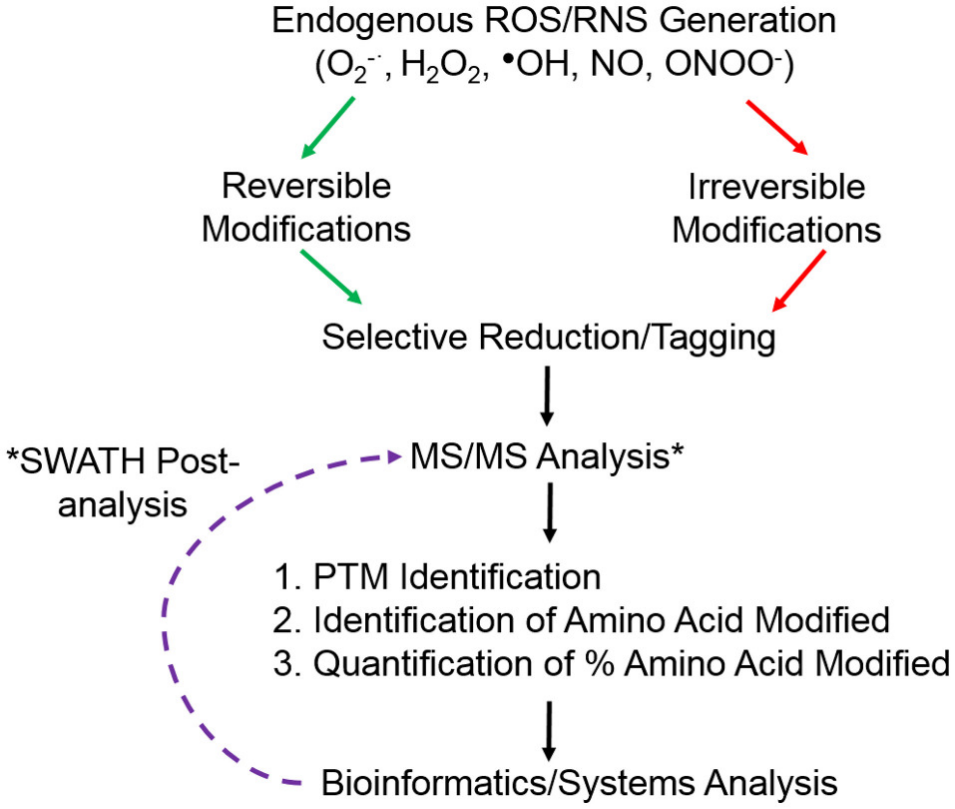
Overview of common redox proteomic approach to identify and quantify ROS/RNS induced protein modifications. ^*^Retrospective analysis of data from SWATH-MS or data independent analysis.

There have been two general approaches in which MS has been applied to proteomics; a discovery proteomic and targeted proteomic approach (Figure 1). The discovery or shotgun proteomic involves proteolytic digestion of a population of proteins and analyzing resulting peptides by MS/MS. It is a global, high throughput approach used for global profiling of systemic perturbations and data analysis allows the identification of proteins that were present in the original protein population. It has a number of limitations due to the complexity of the proteome, namely the most abundant proteins can be identified multiple times and technical replicates can show limited overlap (Malmstrom et al., 2007). If applied to redox proteomics it means that many of the proteins with reactive thiols that are modified to the greatest degree by an oxidant could potentially be less abundant than those modified to a lesser degree and thus not detected. A targeted proteomic or hypothesis-driven approach is used as an analytical tool for structural and molecular studies of a specific protein, where specific peptides are selected for analysis in MS.

The increasing sensitivity and resolution of MS instruments has allowed the characterization of redox induced PTMs, identifying not only the protein susceptible to the modification but also the amino acid residue that is modified. A further goal of redox proteomic approaches is to quantify the proportion of the residue that is modified in the context of the whole protein (Figure 1). Specifically concentrating on Cysteine residues there are a number of approaches that have taken advantage of reversible PTMs whereby initially all free thiols are blocked with an alkylating reagent, such as iodoacetamide or N-ethylmaleimide, followed by the selective reduction of the PTM and subsequent labeling with either another alkylating reagent or a heavy isotope of the initial blocking reagent. Application of a reducing agent, such as dithioreitiol or Tris(2-carboxyethyl)phosphine (TCEP) will reduce all disulfide bonds, while using more specific enzymes or reagents can selectively reduce Cysteine residues that were S-nitrosylated using sodium ascorbate for S-nitrosylation, glutaredoxin for S-glutathionylation, sulfenic acids can be directly labeled with dimedone or reduced using sodium arsenite (Guo et al., 2014). Experimental conditions must be carefully controlled and appropriate controls included for selective reduction to avoid false positive results e.g., the concentration of GSH employed with glutaredoxin needs to be carefully determined as it will have an effect on the specificity of reduction. Subsequent labeling of the newly reduced thiols with alkylating reagents in particular with a biotin moiety attached allows for selective enrichment and MS analysis. However, in order to quantify the proportion of that residue that was modified it is necessary to analyse the initial Cysteine residue in the free thiol state. Redox proteomics has taken advantage of the thiol specificity of the ICAT reagents to not only identify targets of ROS but also to quantify the oxidative thiol modifications on individual proteins. The reactivity and versatility of the ICAT reagents in redox proteomics has been further exploited by the group of Jakob in a technique they termed OxICAT (Leichert et al., 2008). They used the regents to determine the oxidation state of an individual protein thiol in a complex protein mixture, samples are first denatured in a buffer containing a high urea concentration and free thiols are alkylated with the light ICAT reagent. All reversible oxidative thiol modifications are then reduced using the thiol reductant TCEP and newly accessible thiols labeled with the heavy ICAT reagent. Proteins are tryptic digested, labeled peptides affinity purified using the biotin tag on ICAT reagents and analyzed using MS/MS. The ratio of reversibly oxidized and reduced thiol modifications of a particular peptide can thus be determined in one experiment. As the redox proteome equilibrium is constantly changing in response to various stimuli including ROS, this approach provides a large amount of detail regarding the oxidation states of individual proteins at any moment. Combining selective thiol labeling with Isobaric tags for relative and absolute quantification (iTRAQ) also allows the relative quantification of the oxidation state of the Cysteine residue with the advantage that a multiplex approach may be taken (McDonagh et al., 2012).

Nevertheless, with any of these enrichment approaches there is the major disadvantage that information on the overall protein abundance is lost, so although there may be a change in the redox state of a particular Cysteine between two samples, it is not known if the protein itself changes in abundance. To acquire information on protein abundance and the redox state of individual Cysteine residues it is necessary to use sequential labeling of the reduced and reversibly oxidized Cysteine residue with light/heavy isotopes of the alkylating reagent. Combining global label free proteomics together with selective analysis of the ratio of light/heavy labeled Cysteine's allows the relative quantification of the redox state of individual Cysteine residues in the context of the abundance of that protein (McDonagh et al., 2014). However, as with any of the shotgun proteomic approaches mentioned above, it is limited to the detection of the most abundant proteins and peptides. In response to localized ROS/RNS generation only a specific or relatively small proportion of the protein may be modified making it extremely difficult to determine subtle changes in the relative abundance of the modification. The application of targeted MS approaches increases the sensitivity for quantification of the redox state, however it is necessary to have a prior knowledge of the Cysteine containing peptide that is modified for approaches, such as selected/multiple reaction monitoring and parallel reaction monitoring (S/MRM and PRM). This approach has been widely utilized in the study of protein phosphorylation and will provide a more accurate determination of ROS/RNS induced PTMs. One of the first applications of MRM to target modified Cysteine residues used a combination of protein purification, differential Cysteine labeling and MRM to identify the site specific Cysteine oxidation of endogenous p53 (Held et al., 2010).

## Perspective and outlook

Developments in data independent acquisition (DIA) in MS and more recently SWATH (sequential window acquisition of all theoretical fragment ion spectra) where all peptide precursors detected are fragmented (Gillet et al., 2012), will potentially offer higher specificity, reproducibility and dynamic range for the detection of ROS modified peptides and proteins. SWATH combines DIA with targeted analysis and allow post acquisition analysis of data for the identification and quantification of redox modifications. Recently this approach has been combined with affinity purification for the identification of carbonylated residues in rice embryo during seed germination (Zhang et al., 2016). Large numbers of raw data files generated from SWATH and other large scale proteomics studies including detailed experimental conditions are now routinely deposited in public repositories allowing subsequent re-analysis using spectral libraries of previous discovery data to detect and quantify redox modified peptides and proteins. This will allow researchers from all areas to investigate and search data files for specific redox modifications on their proteins of interest. The ongoing developments in both instrument sensitivity and resolution together with more sophisticated bioinformatic tools can potentially allow for the retrospective identification and quantification of specific ROS/RNS induced PTMs. One of the biggest challenges in the analysis of ROS/RNS induced PTMs is the dynamic nature of the modifications in cell signaling and potential cross-talk between redox and non-redox dependent PTMs that regulate protein activity. Including an increased number of potential PTMs combined with targeted post analysis would increase sensitivity and provide a comprehensive overview on the role of ROS induced cellular signaling.

## Author contributions

The author confirms being the sole contributor of this work and approved it for publication.

### Conflict of interest statement

The author declares that the research was conducted in the absence of any commercial or financial relationships that could be construed as a potential conflict of interest.
